# Patient costs incurred by people living with HIV/AIDS prior to ART initiation in primary healthcare facilities in Gauteng, South Africa

**DOI:** 10.1371/journal.pone.0210622

**Published:** 2019-02-11

**Authors:** Natasha Pillai, Nicola Foster, Yasmeen Hanifa, Nontobeko Ndlovu, Katherine Fielding, Gavin Churchyard, Violet Chihota, Alison D. Grant, Anna Vassall

**Affiliations:** 1 Social and Mathematical Epidemiology, London School of Hygiene and Tropical Medicine, London, United Kingdom; 2 Health Economics Unit, School of Public Health and Family Medicine, University of Cape Town, Cape Town, South Africa; 3 Department of Clinical Research, London School of Hygiene and Tropical Medicine, London, United Kingdom; 4 Aurum Institute, Johannesburg, South Africa; 5 Department of Infectious Disease Epidemiology, London School of Hygiene and Tropical Medicine, London, United Kingdom; 6 School of Public Health, University of the Witwatersrand, Johannesburg, South Africa; 7 Africa Health Research Institute, School of Nursing and Public Health, University of KwaZulu-Natal, South Africa; Sefako Makgatho Health Sciences University, SOUTH AFRICA

## Abstract

**Purpose:**

To quantify costs to patients of accessing HIV care prior to ART initiation.

**Materials and methods:**

Using a cross-sectional study design, costs incurred by HIV-positive patients prior to ART initiation were estimated at urban primary healthcare facilities in South Africa. Costs included direct costs, indirect (productivity) costs, carer and coping costs (value of assets sold and money borrowed). The percentage of individual income spent on healthcare was calculated and compared by patient income tertiles and CD4 count strata.

**Results:**

289 patients (69% female, mean age 37 (SD: 10) years, median CD4 317 (IQR: 138–494) cells/mm^3^) were interviewed. The total mean monthly cost of pre-ART care was US$15.71. Indirect costs accounted for $2.59 (16.49%) of this when time was valued using the patient’s reported income. The mean monthly patient costs were $31.61, $12.78, $12.65 and $11.93 for those with a CD4 count <100, 101–350, 351–500 and >500 cells/mm^3^ respectively. The percentage of individual income spent on healthcare was 7.25% for those with a CD4 count <100 cells/mm^3^ and 4.05% for those with a CD4 count >500 cells/mm^3^.

**Conclusions:**

Despite the provision of charge-free services at public clinics, care prior to ART initiation can be costly, particularly for the poor and unemployed. Our study adds to the growing body of evidence that highlights the need to consider policies to reduce the economic barriers to HIV service access, particularly for low income or unwell patient groups, such as improving access to disability grants.

## Introduction

Antiretroviral treatment (ART) initiation when HIV disease is advanced is associated with poor treatment outcomes and high mortality rates [1, 2]. Current international guidelines call for universal test and treat (UTT), ART initiation irrespective of CD4 count [3]. While the health benefits of early initiation of ART and universal test and treat (UTT) are well established, less is known about the economic benefits. Although some studies have documented the patient costs and cost drivers associated with ART, data on the cost to HIV-positive patients prior to starting ART are scarce [4–6].

An understanding of the costs of accessing HIV care prior to ART initiation and the affordability of care seeking is needed to quantify the full benefits of the UTT policy from the perspective of patients. As part of a study conducted in 2013, we assessed the health service utilisation and costs incurred by pre-ART patients in an urban setting in South Africa with high-density housing including informal settlements and high rates of poverty. We examine the direct (out-of-pocket) and indirect (productivity) costs associated with seeking HIV care and explore how differences in valuing patients’ time may influence our cost estimates. Finally, we examine how costs vary by income group and CD4 count.

## Materials and methods

### Study setting

At the time of this study, South African guidelines recommended ART initiation at CD4 count ≤ 350 cells/mm^3^ or WHO clinical stage ≥ 3. Following diagnosis and prior to ART initiation (the pre-ART phase of care), the guidelines recommended that patients be monitored within a ‘Wellness Programme’. This included co-infection screening and treatment, support and counselling, prevention of HIV transmission, family planning and nutrition. Eligible patients were also recommended to receive six-monthly CD4 testing to ensure prompt ART initiation upon reaching the threshold CD4 count [7].

Primary healthcare is provided free-of-charge in the South African public sector. Non-conditional, means-assessed, cash transfers (the disability grant) are available to adults with HIV/AIDS who are unable to work due to mental or physical disability [8]. The disability grant aims to provide social security from poverty associated with illness.

### XPHACTOR interventional cohort study

This study was conducted as part of the XPHACTOR study, a prospective cohort study evaluating a risk-based algorithm to prioritise Xpert MTB/RIF testing among adults attending routine HIV care in South Africa [9]. HIV-positive pre-ART and ART patients were recruited from separate facilities providing HIV care. Pre-ART patients were enrolled in Ekurhuleni district in Gauteng province, South Africa, an urban area with high-density housing, including informal dwellings. The HIV prevalence in this district was estimated to be 14.3% in 2012, higher than the national average of 12.2% [10].

### XPHACTOR economics sub-study

We conducted a cross-sectional patient cost study of HIV-positive patients receiving pre-ART care. Patients were established in care (i.e. not newly diagnosed HIV-positive) and recruited from two primary healthcare facilities. A consecutive sample of participants at enrolment to the ‘XPHACTOR’ study was recruited to the XPHACTOR economics sub-study, until the required sample size was reached. Structured interviews were conducted between June 2013 and March 2014 using an economics study questionnaire (see Supporting Information). The target sample size for this patient cost study was 250 pre-ART patients, limited by logistical constraints.

### Cost estimates

We estimated the direct, indirect, carer, and coping costs associated with reported healthcare provider encounters over the three-month period prior to enrolment in the study, using a purpose designed questionnaire [11, 12]. Direct medical costs included fees for consultation, diagnostic tests and medication from private providers. Direct non-medical costs included out-of-pocket payments for travel and nutritional supplements. Indirect costs included opportunity costs associated with travel time, time spent in healthcare facility and informal carers’ time. Coping costs were estimated from reported money borrowed and assets sold. Detailed questions were asked regarding patients’ income from various sources including formal and informal employment. Costs were stratified by CD4 count data, estimated from the most recent baseline CD4 count available on record at enrolment.

### Data analysis and cost estimation

Data were double-entered using Epidata v3.1 and compared to minimise data-entry errors. Data were analysed in STATA 13 (College Station, Texas). Costs and income were converted from South African Rand (ZAR) to US $ using the average annual exchange rate for 2013, US$1 = ZAR9.66 [13].

Monthly direct ‘out of pocket’ costs (medical and non-medical) were estimated by multiplying the mean cost incurred per visit by the mean number of visits per month. The mean reported number of visits per month was calculated from the number of visits in the three months prior to enrolment [14].

Indirect costs attempt to assign a monetary value to the productivity and economic losses patients and their households experience because of illness or seeking care. Two methods were used to estimate indirect costs. The ‘reported income approach’ used primary data collected by multiplying time spent at clinic visit and travel by the patient’s self-reported hourly wage [14–16]. Self-reported hourly wage was estimated from responses on detailed questions about income sources in the previous three months. Hourly wage rate was estimated from monthly income by assuming 22 days per month (the median number of reported days worked among employed study participants, IQR: 21–26) and 8 hours a day worked. This is based on estimates of weekly hours worked among South Africans [17]. The indirect cost of inpatient stay was calculated by multiplying the number of inpatient nights by daily income to reflect the opportunity cost.

The Human Capital or ‘reported income approach’ has equity implications since it implicitly attaches no value to the time of non-waged workers or those working in the home. In addition, reported income can underestimate the productivity loss to patients, as it excludes unwaged productivity; this is important given the high proportion of low income or unemployment in our study population and among the Southern African HIV/AIDS population [18–20]. Conversely, assigning the average reported income to those who reported no income or low income may overestimate earnings; and may not capture the earning potential of our study population while unwell. The limitations of both approaches are highlighted by Kik et al. [21] and Foster et al. [11]. We therefore used an alternative approach, ‘the minimum wage approach’ as applied by Sinanovic et al. [22]. We valued time cost as a product of time and the minimum legal wage rate for Gauteng, which was $1.61 per hour at the time of this study [11, 23]. This approach assigns a minimum wage to all; and allowed a useful comparison of costs and access to services in the scenario where the earning potential was equal across the study population.

To understand the wider impact of illness, coping costs (the value of assets sold and money loaned) and informal carer costs (calculated as the product of the carer time spent accompanying a patient to a healthcare visit or caring for a patient and the minimum wage rate for Gauteng [20, 24]) were estimated. Costs to patient were calculated as the sum of direct costs and indirect costs. Total costs were calculated as the sum of direct, indirect, carer and coping costs. We explored the differences in cost burden by individual income level and the most recent CD4 count available at time of enrolment (collected from patient records) using the Kruskal-Wallis test.

To highlight the potential for costs due to illness to contribute to a cycle of impoverishment, we compared costs to patient income. We present the (direct and indirect) costs of accessing healthcare as a proportion of monthly individual income. Where patients reported no income, a $1 monthly income was imputed to avoid dividing by zero [11, 25]. However, given that levels of catastrophic costs have been found to be an inconsistent indicator of household impoverishment due to ill health [26, 27], we did not compare the costs against a threshold to express proportion of patients incurring catastrophic costs but rather exploredhousehold coping mechanisms as a proxy for understanding the economic burden of ill health.

### Ethics

The human research ethics committees at the University of Cape Town, University of the Witwatersrand and London School of Hygiene & Tropical Medicine granted ethical approval for the study. Health Department officials and facility managers provided permission to conduct the study in the selected clinics and written informed consent was obtained from respondents.

## Results

### Descriptive statistics

A total of 294 HIV-positive patients were interviewed, of which, five were subsequently discovered to be receiving ART at the time of enrolment and were therefore excluded from the analysis, leaving 289 patients in the final sample. Of the patients interviewed, 69% (200/289) were female and the mean age of the cohort was 37 (SD: 10) years. The key characteristics of the study participants are summarised in Table 1, but briefly, 53% (154/289) had CD4 counts bellow 350 cells/mm^3^ and were eligible for ART at the time of the study; 22% (64/289) had CD4 counts between 351 and 500 cells/mm^3^ and 25% (71/289) of patients had CD4 counts higher than 500 cells/mm^3^. The median time since HIV diagnosis was 12 months. In terms of socio-economic status, 41% (118/289) of participants reported living on less than $31.00 per month, which is below the World Bank poverty threshold of $38.02 per month [28]. The mean reported hourly income was estimated at $0.92. In contrast, only 20% (57/289) of patients reported receiving a government grant as their primary source of income (e.g. unemployment, child or care dependency grants) with 4% (4/57) of these receiving the disability grant. Approximately half of all respondents at 48% (138/289) reported one or more symptoms suggestive of tuberculosis (TB), 17% (50/289) of patients were taking isoniazid, 43% (123/289) were taking co-trimoxazole and 6% (16/289) both isoniazid and co-trimoxazole. In terms of accessing health services, 50% (145/289) of patients attended the public clinic at least monthly and 90% (275/289) at least once in the previous three months.

**Table 1:** Description of study participants (n = 289).

Descriptor	Number	Statistic
Mean age in years (standard deviation)	38	(10)
Gender, female (%)	200	(69%)
*Median time since HIV diagnosis (months*, IQR) (n = 288)**	*12*	*(1–76)*
*CD4 cell count in cells/mm*^*3*^ *(%)*		
0–100	49	(17%)
101–350	105	(36%)
351–500	64	(22%)
>500	71	(25%)
Number of symptoms suggestive of TB*		
None	151	(52%)
1–2	113	(39%)
3–4	25	(9%)
*Education (%) (n = 288)*		
Primary school or lower	13	(5%)
Secondary school	35	(12%)
High school	135	(47%)
School leaver’s certificate	105	(36%)
*Dwelling (%) (n = 286)*		
House/flat	169	(58%)
Room/flatlet	81	(28%)
Informal/other	39	(13%)
*Water Source (%)*		
Piped water inside dwelling	126	(44%)
Piped water inside yard	151	(52%)
Piped water on community stand	12	(4%)
*Marital status (%) (n = 288)*		
Single never married	151	(52%)
Married/co-habiting	110	(38%)
Divorced/separated/widowed	27	(9%)
*Primary source of income (%)*		
Formal employment	70	(24%)
Informal employment/odd jobs	46	(16%)
Self-employment	33	(11%)
Government grants as the primary source of income	57	(20%)
Other income	13	(4%)
No income source reported	68	(24%)
*Mean number of monthly visits to healthcare provider (median*, *IQR)*		
Public provider (n = 281)	1.05	(0.35, 0–2)
Private provider (n = 76)	0.38	(0.75, 0–3)
*Mode of travel to public clinic (%) *		
Walked	163	(57%)
Bus/taxi	111	(39%)
Car	14	(5%)

* Any of cough, fever, night sweats and/or unintended weight loss

** Time from HIV diagnosis to date of study enrolment interview. IQR interquartile range

**Fig 1:**
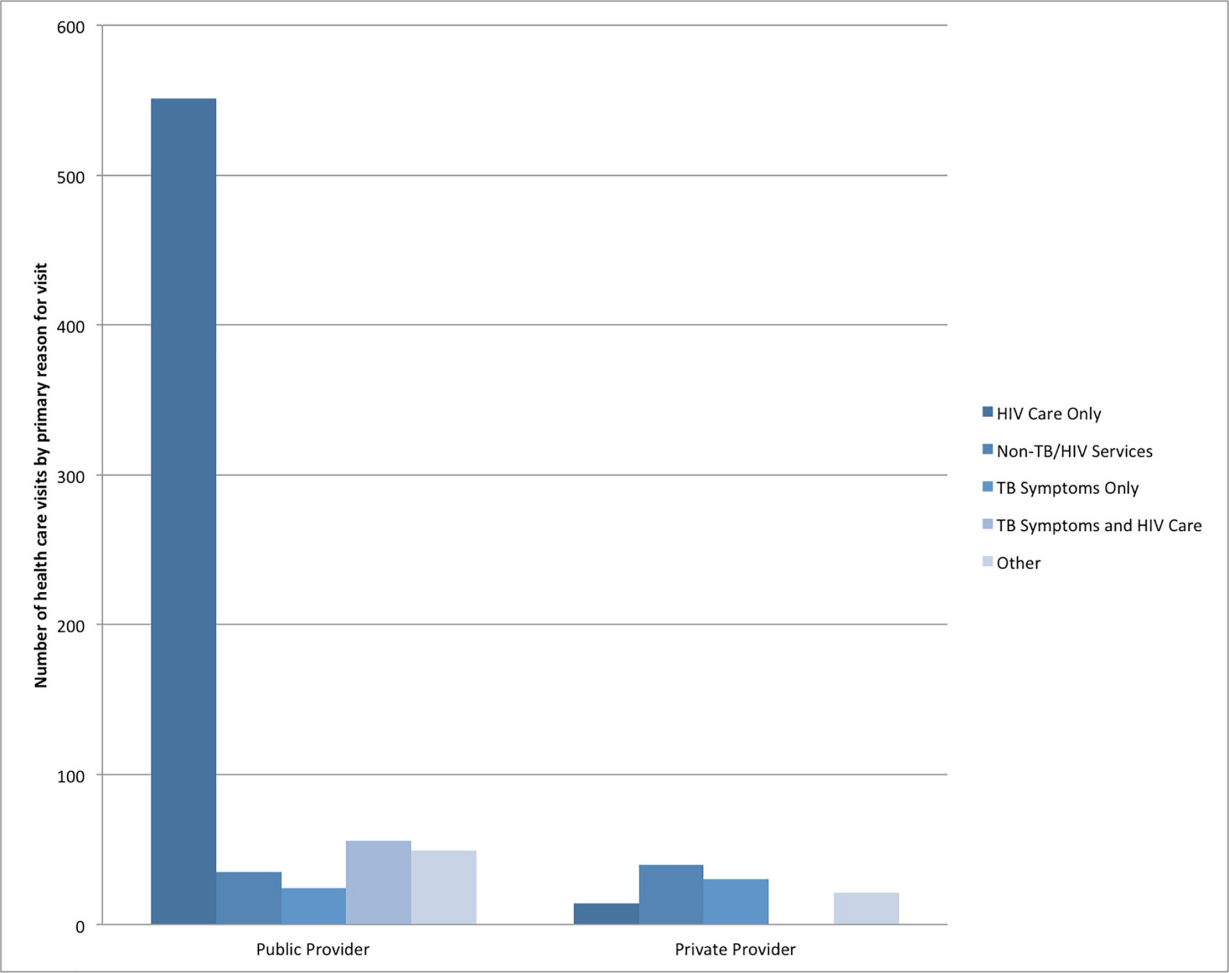
Reasons for seeking healthcare among pre-ART patients. Respondents were asked about their health service utilisation and reasons for service utilisation in the last 3 months; this was based on the responses of 277 respondents (a total of 715 visits). 76 (105 visits) respondents utilised services at a private provider.

Fig 1 describes the frequency of health care visits and reasons for seeking healthcare among pre-ART patients in the three months prior to study enrolment. The main reason for seeking care from the study clinic was HIV care. Those seeking care from a private provider cited non-TB/HIV care and TB symptoms as the main reasons for attending. The majority of patients sought care from the clinic where they were interviewed and 96% (277/289) reported that this was their usual public clinic. The main mode of transport to the public clinic was walking (60% of patients) suggesting good availability of health services.

### Direct and indirect costs

Table 2 summarises the monthly direct -, carer -, coping -, indirect-, and total costs as well as the percentage of income spent on care at different levels of income. Total monthly costs varied depending on how time was valued. The mean monthly cost of pre-ART care incurred by the patients alone (patient-only costs), was $9.16 when applying the reported income approach. This value rose to $12.58 using the minimum wage approach. Patient-only costs were primarily driven by the cost of time spent during public clinic visits ($2.00 using the reported income approach and $4.75 using the minimum wage approach), the out-of-pocket cost of private doctor care ($1.78) and the purchase of dietary supplements ($3.53) per month. The incremental mean monthly cost of care when including carer costs was $3.42 and $15.71 respectively when applying the reported income approach and the minimum wage approach. The ‘reported income approach’ showed that the highest income group also incurred more direct costs, though this represented a smaller percentage of their income.

**Table 2:** Health service utilisation and costs by income categories, monthly (mean)*.

									*Reported income approach*			*Minimum wage approach*					
Individual Income tertiles (n)	Direct Costs	Frequency of health service visits	Direct costs as % of income	Carer Costs	Money Borrowed		Value of Assets Sold		Indirect Costs	Cost to Patient	Cost to patient as % of income^†^	Indirect cost	Cost to Patient	Cost to patient as % of income	Total costs^Ψ^ as % of income adjusted for those with no income	Total cost (reported income approach)^Ψ^	Difference in total cost between reported income and minimum wage approaches
**$ < 9 (99)**	$5.58	0.89	0.0%	$6.45	$1.36		$0.46		$0.00	$5.58	0.0%	$6.64	$12.22	0.0%	1222.2%	$18.67	$6.64
**$ 9–181 (94)**	$5.56	0.94	8.9%	$0.49	$1.19		$0.70		$1.22	$6.78	10.0%	$5.19	$10.76	5.9%	17.3%	$11.25	$3.98
**$ 181–1242 (96)**	$8.58	1.01	2.9%	$2.29	$8.90		$0.25		$6.61	$15.19	4.9%	$6.16	$14.74	0.2%	5.1%	$17.03	-$0.45
**Total (289)**	$6.57	0.95	3.8%	$3.13	$3.81		$0.47		$2.59	$9.16	4.9%	$6.01	$12.58	2.1%	426.0%	$15.71	$3.42
p-value ***	0.066	0.187	<0.001	0.292	0.151		0.629		<0.001	<0.001	<0.001	0.732	0.347	<0.001	<0.001	0.333	

*1 US$ = R9.66 (2013); Reported income approach: Indirect costs calculated from individual productive income. Minimum wage approach: Indirect costs calculated by multiplying minimum wage for Gauteng by waiting plus travel time.

^**Ψ**^ Total cost: Sum of direct, indirect (reported income approach), carer and coping costs.

***Kruskal-Wallis test examining differences in costs by income tertile

Coping mechanisms used to deal with the cost burden also varied between income groups (Table 2). Those with higher income levels borrowed more money while those with less income sold assets. Some participants (14%) borrowed money in order to cope with pre-ART care costs, with more than half of those who borrowed money (69%) having to pay interest on money borrowed.

Monthly income and patient costs were compared between CD4 count groups (Table 3). Patients with low CD4 counts had the lowest income and the highest costs. When accounting for carer and indirect costs, those with the lowest CD4 count (<100 cells/mm^3^) incurred the highest cost with a total monthly cost of $31.61 compared to the sample mean total monthly cost of $15.71 (P = 0.12 for difference in total costs by CD4 stratum, Kruskal Wallis test). Income was lower in this group with the mean monthly income of $115.41 compared to the study population average of $144.62 and $176.97 for those with a CD4 count between 350 and 500 cells/mm^3^. When comparing indirect cost approaches, the lowest CD4 count group incurred the highest difference in monthly patient only costs: $6.26 ($12.79 vs $19.05 using reported income and minimum wage approaches respectively). Using two methods of valuing patients’ time (indirect costs) highlights that the burden of medical expenses is most likely to impact those with low income.

**Table 3:** Health service utilisation and costs by CD4 count, monthly (mean)*.

								*Reported Income approach*			*Minimum wage approach*					
*CD4 Cell Count (n)*	*Monthly reported Individual Income*	*Healthcare visits in 1 month period*	*Direct Costs*	*Direct costs as % of income*	*Carer Costs*	*Money Borrowed*	*Value of Assets Sold*	*Indirect Costs*	*Cost to Patient*	*Cost to patient as % of income*	*Indirect Costs*	*Cost to Patient*	*Cost to patient as % of income*	Total cost ^Ψ^ as % of income adjusted for those with no income	Total Cost ^Ψ^ (reported income approach)	Difference in cost to patient between indirect cost approaches
***0–100*** ***(49)***	*$115*.*41*	*0*.*90*	*$9*.*05*	*5*.*71%*	*$12*.*56*	*$0*.*26*	*$0*.*80*	*$3*.*74*	*$12*.*79*	*7*.*25%*	*$10*.*00*	*$19*.*05*	*16*.*51%*	*806*.*65%*	*$31*.*61*	*$6*.*26*
***101–350*** ***(105)***	*$134*.*23*	*0*.*91*	*$6*.*45*	*3*.*92%*	*$0*.*93*	*$0*.*16*	*$5*.*12*	*$2*.*58*	*$9*.*03*	*4*.*93%*	*$5*.*40*	*$11*.*85*	*8*.*83%*	*407*.*55%*	*$12*.*78*	*$2*.*82*
***351–500*** ***(64)***	*$176*.*97*	*0*.*95*	*$5*.*35*	*2*.*94%*	*$2*.*10*	*$0*.*38*	*$5*.*45*	*$2*.*30*	*$7*.*66*	*3*.*82%*	*$5*.*10*	*$10*.*45*	*5*.*90%*	*257*.*97%*	*$12*.*56*	*$2*.*80*
***>500*** ***(71)***	*$150*.*98*	*1*.*019*	*$6*.*14*	*3*.*20%*	*$0*.*81*	*$1*.*14*	*$2*.*48*	*$2*.*07*	*$8*.*22*	*4*.*05%*	*$4*.*98*	*$11*.*12*	*7*.*36%*	*342*.*03%*	*$11*.*93*	*$2*.*91*
***Total*** ***(289)***	*$144*.*62*	*0*.*95*	*$6*.*57*	*3*.*83%*	*$3*.*13*	*$0*.*47*	*$3*.*81*	*$2*.*59*	*$9*.*16*	*4*.*86%*	*$6*.*01*	*$12*.*58*	*8*.*70%*	*426*.*00%*	*$15*.*71*	*$3*.*42*
***p-value*** ***	*0*.*178*	*0*.*532*	*0*.*290*	*0*.*922*	*0*.*149*	*0*.*508*	*0*.*155*	*0*.*420*	*0*.*402*	*0*.*924*	*0*.*479*	*0*.*174*	*0*.*040*	*0*.*040*	*0*.*121*	

*1 US$ = R9.66 (2013); Reported income approach: Indirect costs calculated from individual productive income. Minimum wage approach: Indirect costs calculated by multiplying minimum wage for Gauteng by waiting plus travel time. Total costs as a % of income adjusted for those with no income: percentage of total individual income spent on pre-ART care, income set to 1 for those with no reported monthly income.

^**Ψ**^Total cost: Sum of direct, indirect (reported income approach), carer and coping costs.

***Kruskal-Wallis test examining differences in costs by CD4 count level

The percentage of individual income spent on healthcare was highest for the lowest CD4 count group (7.3% and 16.5% for the reported income and minimum wage approaches respectively).

## Discussion

This study demonstrates that despite the provision of free health services at public clinics in South Africa, pre-ART healthcare can be costly, particularly for low income and low CD4 count groups. Time costs represent a large proportion of total costs that may impact household economic status due to frequent healthcare visits. The importance of time costs to patients as a risk to care are highlighted in the literature [29–32].

### Study findings in the context of existing evidence

This study is supported by published literature on the patient costs of HIV care in other African settings. Vassall et al. [33] explored the patient costs incurred by TB/HIV patients receiving ART in Ethiopia and stressed the burden of indirect costs borne by patients when seeking care. Other studies report similar results, but different patterns of cost drivers. Miller et al. found that transport costs and time commitments pose significant risks to accessing ART in two NGO run clinics in the Limpopo and Gauteng province in South Africa[34]. In their Zambian study, Fox et al. (2010) found that perceived financial and logistical challenges strongly deterred patients from accessing ART, even when it was available free-of-charge [35].

Our study adds to previous findings on care-seeking behaviour and corresponding pre-ART patient costs. Chimbindi [36] and Rosen et al. [37] explored the costs of accessing pre-ART and ART care in South Africa, but Rosen did not disaggregate results by ART status. Chimbindi et al. reported higher total monthly costs for pre-ART care ($23.42) but did not explore how levels of immunosuppression influenced costs. Compared to this study, Chimbindi et al. found that a higher proportion of pre-ART patients in a rural community sought care from traditional healers and healthcare providers outside of the study clinic [36]. In contrast, Rosen et al. [37] found that a relatively small proportion of urban patients receiving HIV care sought care outside the study clinics. In addition, they reported lower average costs per week of care received outside the study clinic $2.41 (2007 $1 = ZAR 7.05) but a higher proportion of spending on dietary supplements ($16.31 on average per week) [13]. This may reflect that pre-ART patients have a high demand for healthcare and may seek care from non-public care providers due to factors such as comparative quality of care, shorter waiting times, attitudes, beliefs and information. Once ART is initiated, the literature suggests that people seek care from traditional healers and private providers less [38].

### Methodological approaches to valuing patient time

The different methodological approaches we used to value indirect costs may influence policy implications, as they represent different views on ‘economic burden’. By failing to value the indirect cost burden borne by the unemployed and poor, economic evaluations reflecting productivity losses only, may not fully reflect the economic burden of healthcare as perceived by patients and their community. This is particularly disconcerting since the unemployed and poor might be disproportionately affected by the opportunity cost of job seeking, education, childcare or household commitments. While the minimum wage approach highlights the value of time for the unemployed or low-income groups, it conversely undervalues time for those in employment, and may underestimate productivity losses. This approach may also not completely acknowledge the reduced access to services that higher income groups may face when seeking care compromises their work commitments.

Our analyses highlight the influence these approaches to valuing indirect costs have on patient cost estimates. Applying an equal wage to the entire study population acknowledges the opportunity and productivity costs faced by those who do not earn a monthly wage with regard to the household child-care duties as well as educational and job-seeking activities (3). The human capital approach in cost of illness studies conceptualises time from the patient’s perspective as part of their human capital. We recognise that neither approach is perfect and thus present the results of both approaches for comparison.

### Strengthening systems to reduce the poverty impact of HIV care

This study pre-dated the UTT policy, undertaken when South African guidelines recommended ART initiation at CD4 count ≤ 350 cells/mm^3^ or WHO clinical stage ≥ 3. It highlights the need for policies that protect people from the poverty impact of HIV care. Patients reported coping with the cost of their care by borrowing money or selling assets. These coping costs could have a long-term impact on the patient and their household’s wealth and economic status. We found weak evidence that people with low CD4 counts had higher costs of care prior to ART initiation and lower income levels (Table 3). The ratio of cost to individual income was highest for the lowest CD4 count group (7.3% and 16.5% for the reported income and minimum wage approaches respectively) (Table 3). Moshabela et al. note that concurrent use of public, private and traditional healthcare pathways prior to ART initiation contribute to poor retention in care, high patient costs and delays in treatment initiation, and people taking ART are less likely to seek care from alternative providers [39]. The high frequency of clinic follow-up for some patients may have contributed to the high indirect costs presented here. This underlines the importance of reducing the frequency of clinic visits wherever possible.

Only four patients in this study reported an income from disability grants, despite disability grants being available to a larger proportion of our study population [26]. This could be explained by the challenges to accessing disability grants reported elsewhere [8]. Improvements to the process of identifying eligible patients and how they access disability grants might reduce the poverty impact of HIV in South Africa.

### Study strengths and limitations

The methodological approaches to valuing patients’ time presented here were used both for practical measurement reasons and for their comparability as good estimates of productivity loss. Since a high proportion of our study group reported earning nothing or less than the minimum wage in the three months prior to study enrolment, we hoped to limit the risk of double-counting reduced earning potential due to illness. In addition, since a minority of the study population earned higher than the minimum wage ($1.61 per hour in Gauteng [23]) the minimum wage approach provided a pragmatic estimate of maximum earning potential.

We estimated the costs incurred by pre-ART patients attending public healthcare services. Since we recruited from those already in care, we were less likely to recruit patients who seek care intermittently. Our cost estimates may therefore be skewed towards the higher end, particularly at one study clinic where prophylactic isoniazid and co-trimoxazole were dispensed monthly. Respondents were recruited as part of an interventional study in health facilities and were asked about their individual income as opposed to household income. This therefore limits our ability to explore how household-level consumption may have shifted in response to the costs incurred in accessing healthcare leading us to underestimate the impact on households. Conversely, the ratio of cost to individual income could overestimate the poverty impact of costs in households with more than one source of income or a source of income not captured by our questionnaire. Social desirability bias could also result in patients over-reporting income. We used detailed questions about distinct categories of income to mitigate against this. Social desirability bias may have also resulted in under-reporting of visits to traditional health practitioners. We explored patient coping mechanisms such as borrowing money to pay for health-related expenses and patient carer costs, thereby assessing the economic burden of ill-health more broadly than the household.

## Conclusion

Implementation of international guidelines that call for UTT requires an understanding of the demand-side risks and challenges to accessing pre-ART care. Despite the provision of charge-free services at public clinics, care prior to ART initiation can be costly, particularly for the poor and unemployed. Our study adds to the growing body of evidence that highlights the need to consider policies to reduce the economic barriers to HIV service access, particularly for low income or unwell patient groups, such as improving access to disability grants.

## Supporting information

S1 Table Appendix 1: Monthly income, costs, money borrowed, and assets sold by CD4 count (mean, median and standard deviation). (DOCX)
