# Supplementary material for: Patient costs incurred by people living with HIV/AIDS prior to ART initiation in primary healthcare facilities in Gauteng, South Africa
Source: PLoS One. 2019 Feb 11;14(2):e0210622. doi: 10.1371/journal.pone.0210622 (PMC6370193; doi:10.1371/journal.pone.0210622)
Supplement: S1 Table — (DOCX) [file pone.0210622.s001.docx]

**Appendix 1: Monthly income, costs, money borrowed, and assets sold by CD4 count (mean, median and standard deviation)**

| **CD4 Count** | **Monthly reported individual Income** | | | **Direct Costs** | | | **Indirect cost** | | | **Carer Costs** | | | **Money Borrowed** | | | **Value of Assets sold** | | |
| --- | --- | --- | --- | --- | --- | --- | --- | --- | --- | --- | --- | --- | --- | --- | --- | --- | --- | --- |
|  | **Mean** | **Median** | **Standard deviation** | **Mean** | **Standard deviation** | **Range** | **Mean** | **Median** | **Standard deviation** | **Mean** | **Median** | **Standard deviation** | **Mean** | **Median** | **Standard deviation** | **Mean** | **Median** | **Standard deviation** |
| **0-100** | $115.41 | $60.04 | $151.82 | $9.05 | $3.45 | $13.46 | $3.74 | $0.00 | $10.08 | $12.56 | $0.00 | $67.77 | $0.80 | $0.00 | $3.49 | $0.26 | $0.00 | $1.82 |
| **101-350** | $134.23 | $62.11 | $190.55 | $6.45 | $3.45 | $7.44 | $2.58 | $0.00 | $5.70 | $0.93 | $0.00 | $4.43 | $5.12 | $0.00 | $27.95 | $0.16 | $0.00 | $1.68 |
| **351-500** | $176.97 | $110.25 | $189.44 | $5.35 | $1.69 | $10.95 | $2.30 | $0.93 | $3.87 | $2.10 | $0.00 | $10.12 | $5.45 | $0.00 | $22.70 | $0.38 | $0.00 | $3.02 |
| **>500** | $150.98 | $103.52 | $180.94 | $6.14 | $3.31 | $8.24 | $2.07 | $0.33 | $4.23 | $0.81 | $0.00 | $5.19 | $2.48 | $0.00 | $12.56 | $1.14 | $0.00 | $6.87 |
| **Total** | $144.62 | $72.46 | $182.17 | $6.57 | $3.45 | $9.71 | $2.59 | $0.00 | $6.04 | $3.13 | $0.00 | $28.64 | $3.81 | $0.00 | $20.89 | $0.47 | $0.00 | $3.90 |
